# Rare Form of Intestinal Obstruction—Operation—Cure

**Published:** 1879-05

**Authors:** 


### EXCERPTA.

Rare Form of Intestinal Obstruction—Operation—Cure.—At a recent meeting of the London Clinical Society, Mr. Bellamy read notes of a case of rare form of intestinal obstruction, due to invagination of the small intestine in the first part of the rectum; gastrotomy; recovery. The patient was a pale, delicate woman, aged 34, who came under care on February 16, 1879, with symptoms of obstruction of nine days' standing. She had an inguinal hernia on the left side, for which she wore a truss, which was left off a short time previously. She was subject to habitual constipation, and on three occasions the retention of fecal matter had given rise to very serious symptoms, which, however, always yielded to ordinary measures. On admission, a hard swelling was felt in the left iliac fossa in the region of inguinal canal and sigmoid flexure. There was intense pain over lower part of abdomen, and her eructations smelt stercoraceous. On examination of rectum by the entire hand, under chloroform, Mr. Bellamy found that he could not get his fingers past the lower end of the sigmoid flexure, and that it seemed to be filled up and constricted by some intra-abdominal stricture or protrusion through the separated softened muscular fibres of the rectum. Deferring operation for a time, the patient became much worse, vomiting stercoraceous, and on the evening of the 17th Mr. Bellamy proceeded to operate, under strict antiseptic precautions. Thinking there might be some involution of small intestine, perhaps from hernia into rectum, as mentioned by Linhart, or a hernia reduced *en masse*, he cut down on the external ring, exposed it, and, passing the finger into inguinal canal, found no gut there, but felt the sigmoid flexure greatly distended. Then, enlarging the opening, and introducing the entire hand into abdominal cavity, he found the posterior uterovesical fold of peritoneum greatly developed, and also a bundle of small intestine lying below it and tucked into the anterior wall of the rectum. In addition to this, he felt what appeared to be bands of organized lymph stretching across the first part of rectum, probably due to some earlier inflammation in the same locality, rendering reduction per anum impossible. Again, with the entire right hand in the rectum and the left in the pelvic cavity, he broke down adhesions, and, by gradual manipulation, reduced the prolapsed bowel. Very shortly after, flatus escaped, and within twelve hours a most copious evacuation occurred, affording immense relief. The patient progressed favorably until the 22d, and had not a bad symptom of any sort; but some delirium came on, and she tore off her antiseptic dressings. The delirium was, however, subdued by subcutaneous injection of morphia and by chloral, and at the time of the meeting the wound was looking quite healthy, and she was out of danger.—*Lancet.*

